# Anterolateral ligament reconstruction in addition to primary double-bundle anterior cruciate ligament reconstruction for grade 3 pivot shift improves residual knee instability during surgery

**DOI:** 10.1186/s40634-021-00369-4

**Published:** 2021-07-19

**Authors:** Yusuke Kawanishi, Makoto Kobayashi, Sanshiro Yasuma, Hiroaki Fukushima, Jiro Kato, Atsunori Murase, Tetsuya Takenaga, Masahito Yoshida, Gen Kuroyanagi, Yohei Kawaguchi, Yuko Nagaya, Hideki Murakami, Masahiro Nozaki

**Affiliations:** 1 Department of Orthopedic Surgery, Nagoya City University Graduate School of Medical Sciences, 1-Kawasumi, Mizuho-Cho, Mizuho-Ku, Nagoya, 467-8601 Japan; 2 Kasugai Joint & Sports Orthopedic Clinic, Kasugai, Japan

**Keywords:** Pivot-shift test, Anterior cruciate ligament, Anterolateral ligament, Quantitative measurement, Inertial sensor

## Abstract

**Purpose:**

High-grade pivot shift in the anterior cruciate ligament (ACL) injured knee is a risk factor for postoperative residual pivot shift. Procedures in addition to ACL reconstruction such as anterolateral ligament (ALL) reconstruction have been performed for patients with a high-risk of residual pivot shift. The aim of this study was to investigate the effect of the addition of ALL reconstruction to primary double-bundle ACL reconstruction in patients with preoperative high-grade pivot shift to improve stability as evaluated by quantitative measurement.

**Methods:**

Patients with ACL injuries who showed preoperative grade 3 subjective pivot shift and who underwent primary double-bundle ACL reconstruction combined with ALL reconstructions were retrospectively enrolled. Anterior tibial translation (ATT) in the Lachman test, and acceleration and external rotational angular velocity (ERAV) in the pivot shift were measured as quantitative values. Quantitative values before surgical intervention for ACL-injured knees (ACLD) and uninjured contralateral knees (intact), after temporary fixation of the isolated ACL grafts (ACLR), and subsequently after temporary fixation of both ACL and ALL grafts (ACLR + ALLR) were measured with the patient under general anaesthesia.

**Results:**

In total, 18 patients were included. The ATT was lower in ACLR and ACLR + ALLR than in intact (*P* = .008 and .005), while there was no significant difference between ACLR and ACLR + ALLR (*P* > .05). The acceleration of ACLR + ALLR was lower than that for ACLR (*P* = .008), while there was no significant difference between intact and ACLR or ACLR + ALLR (*P* > .05). The ERAV of ACLR was higher than that of intact (*P* < .001), while that of ACLR + ALLR was lower than that of ACLR (*P* < 0.001), and there was no significant difference in ERAV between intact and ACLR + ALLR (*P* > 0.05).

**Conclusion:**

According to quantitative assessment of the pivot shift, the addition of ALL reconstruction to primary double-bundle ACL reconstruction improved residual knee instability and restored knee stability during surgery. Combination of ALL reconstruction with primary double-bundle ACL reconstruction was effective for patients with ACL injuries exhibiting a preoperative grade 3 subjective pivot shift.

**Level of evidence:**

IV

## Introduction

The anterior cruciate ligament (ACL) is frequently injured in athletic populations. ACL reconstruction has been considered a successful treatment for controlling anteroposterior and rotational stability, evaluated using the Lachman test and pivot-shift test, respectively. However, instability in the pivot-shift test, which correlates with functional outcomes [2], remains in some patients even after ACL reconstruction. Several studies have reported risk factors for residual pivot shift [13, 30] including preoperative high-grade pivot shift.

Recently, additional procedures to ACL reconstruction have been reported. These include anterolateral ligament (ALL) reconstruction [27], anterolateral tenodesis [25] and anterolateral structure augmentation [29] for patients with risk factors, such as revision cases [16], young patients [27], pivoting sports [27], athletes [8], chronic ACL tears [9], hyperlaxity patients [10], and preoperative grade 3 pivot shift [28]. In particular, the ALL, which consists of the anterolateral complex of the knee and has been reported to be a secondary stabiliser to the ACL [7], provides rotational control of the knee. Previous biomechanical studies have shown that the addition of ALL reconstruction to ACL reconstruction for ACL and ALL deficient knees improves rotational instability [22, 31]. Moreover, combined ACL and ALL reconstruction has been reported as an effective approach for lowering the rate of graft failure when compared to ACL reconstruction alone [8, 10, 27]. Double-bundle ACL reconstruction has been also reported to be associated with superior knee stability (both anterior and rotational stability) than single-bundle reconstruction, while subjective scorings have showed no statistical differences [18]. Few studies have investigated the effects of combined double-bundle ACL and ALL reconstruction or the quantitative measurement of instability at the time of additional ALL reconstruction [29].

Several quantitative measurements for instability in the pivot-shift test and rotational instability have been reported [15, 17] and have indicated that quantitative evaluation of instability has better utility than subjective grading alone. Among the quantitative methods proposed is the use of an inertial sensor device, composed of a 3-axis accelerometer and gyroscope, which is small, portable, and non-invasive [20]. This device is useful to evaluate knee instability in clinical settings.

The purpose of the present study was to investigate the impact of the addition of ALL reconstruction to primary double-bundle ACL reconstruction for patients with preoperative high-grade pivot shift, in terms of improving instability by quantitative measurement. The hypothesis was that additional ALL reconstruction improves residual instability in the pivot shift after isolated ACL reconstruction as evaluated by an inertial sensor.

## Methods

### Inclusion in the study

This was a retrospective study. Data were collected at our institution between March 2016 and March 2020. Patients with ACL injuries exhibiting preoperative grade 3 subjective pivot shift, based on the International Knee Documentation Committee Grading (i.e., equal: grade 0; glide: grade 1; clunk: grade 2; gross: grade 3) under anaesthesia, and who underwent double-bundle ACL reconstructions combined with ALL reconstructions were considered eligible for this study. Patients with meniscal injuries were not excluded, regardless of whether or not complete repair was performed in operation. We excluded patients who had prior injuries or surgeries to the contralateral or involved knees. Eighteen patients were included in the final sample. The knees of the included patients had neither severe osteoarthritis (International Cartilage Repair Society grade 3 or 4) nor concomitant ligament injuries requiring reconstruction.

This study was conducted in accordance with the Declaration of Helsinki and was approved by the Institutional Review Board (protocol number: 60–18-0154). Written informed consent was obtained from all patients.

### Surgical technique

All ACL and ALL reconstruction procedures were performed by a senior orthopaedic surgeon, who performs more than 100 cases of ACL reconstruction per year. For patients with high activity levels, additional ALL reconstructions were performed if they had at least one of the following: (1) preoperative grade 3 subjective pivot shift (without anaesthesia in an outpatient consultation room or under anaesthesia in the operating room), (2) revision surgeries, or (3) prior injuries to the contralateral knees. Patients with criteria (2) and/or (3) were not included in this study. An arthroscopic procedure was performed through anteromedial, anterolateral, and medial accessory portals. The status of the ACL injury, meniscus, and cartilage was confirmed arthroscopically. Ramp lesions were addressed in every case by using an intercondylar view and probing through the posteromedial capsule. Meniscal injuries were repaired unless they included degenerative tears in which case partial meniscectomy, or no treatment was performed.

#### Graft preparation

The semitendinosus and gracilis tendon were harvested in the involved knee. Two double-stranded grafts from a semitendinosus tendon for double-bundle ACL reconstruction were created at more than 5.0 mm diameter. A double-stranded graft from a gracilis tendon for ALL reconstruction was created at more than 4.5 mm diameter and 100 mm length.

#### Creation of bone tunnels

**Fig. 1:**
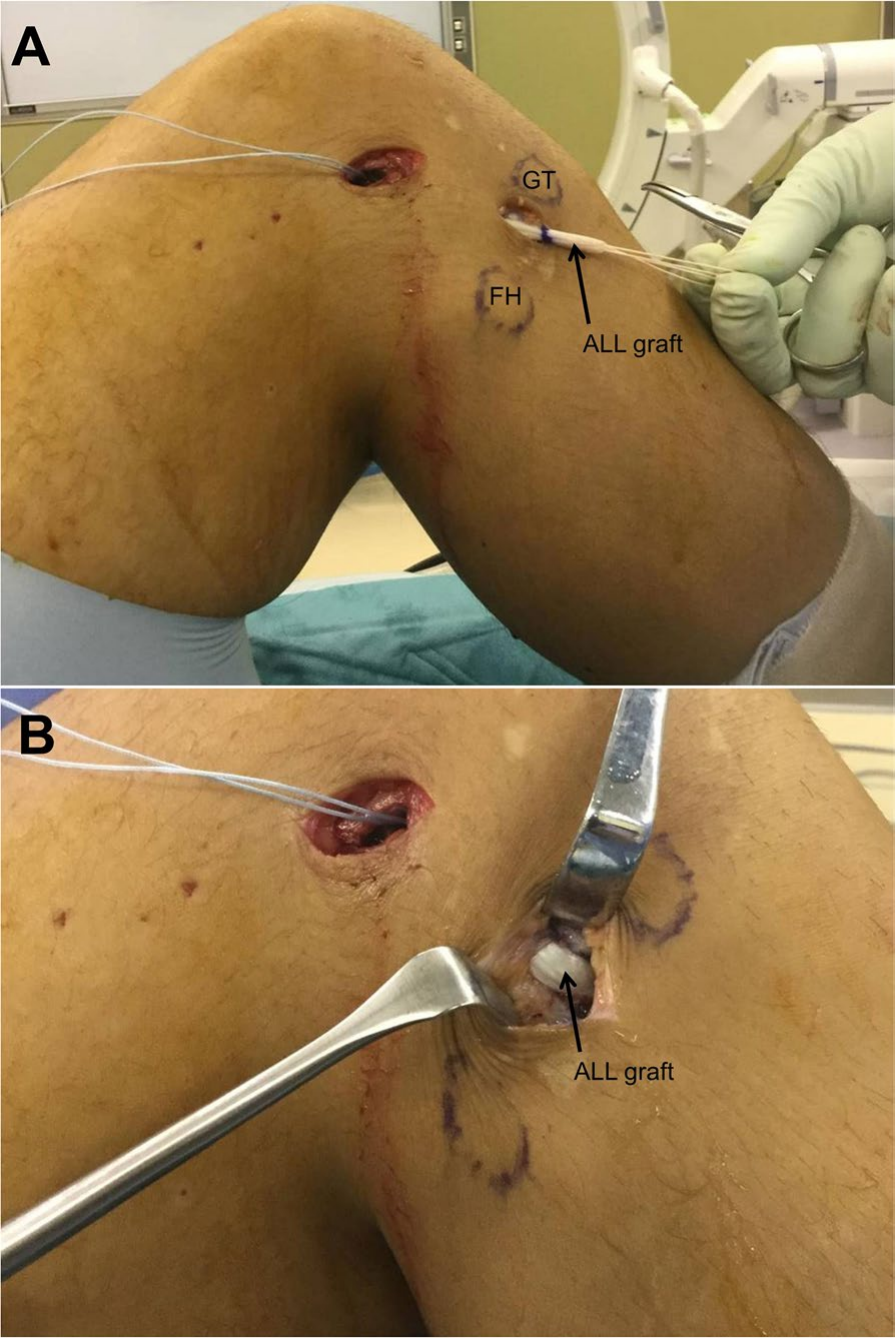
Procedure for anterolateral ligament reconstruction. The view of lateral aspect of a right knee, placed supine during procedure for anterolateral ligament (ALL) reconstruction. **(A)** The femoral tunnel is created posterior and proximal to the lateral epicondyle with a minimal incision. The tibial tunnel is created with another incision from the mid-point between the fibular head and Gerdy’s tubercle. The ALL graft is passed under the iliotibial tract and superficial to the lateral collateral ligament aspect of the tibia, after fixation to the femoral tunnel. **(B)** The ALL graft is passed through the tibial tunnel. GT, Gerdy’s tubercle; FH, fibula head

The procedure of ACL reconstruction was performed according to a previous report [13]. To create the femoral tunnel of the ALL, a minimal incision was made at the lateral side of the knee (Fig. 1A and B). The iliotibial tract was split and retracted to expose the lateral epicondyle. The femoral tunnels were made at the points posterior and proximal to the lateral epicondyle [1, 12, 14]. The tibial tunnel of the ALL was created with a minimal incision from the midpoint between the fibular head and Gerdy’s tubercle [1] (Fig. 1A).

#### Graft fixation

Adjustable loop suspension devices were used for femoral fixation of the ACL grafts and a bioabsorbable interference screw was used for femoral fixation of the ALL graft. The ALL graft was passed under the iliotibial tract and was superficial to the lateral collateral ligament aspect of the tibia through the tibial tunnel [7] (Fig. 1A and B). After femoral fixation, at least 20 flexion–extension cycles (0°-120° of flexion) were performed to prevent graft elongation after tibial fixation. For quantitative measurements of instabilities, temporary fixations of the ACL grafts were performed at 30° of flexion under 40 N for the anteromedial bundle and at 0° of flexion under 30 N for the posterolateral bundle, while temporary fixation of the ALL graft was performed at 20° of flexion under 20 N in the neutral rotational position [12]. Each graft was fixed under tensioning using the ligament tensioner (Ai-Medic, Japan) by clamping just above the tibial cortex using forceps (for example, Pean or Kocher) in the incision. Temporary fixations were performed, following all meniscal procedures such as repairs or resections. After temporary fixation was completed, quantitative measurements were performed. After all measurements were completed, definitive fixations at the tibial sides were performed using staples and bioabsorbable interference screws for ACL grafts, while a bioabsorbable interference screw was used for ALL grafts.

### Quantitative measurements of knee instability

All quantitative measurements of instability were carried out under general anaesthesia by the senior surgeon. Quantitative values just before the surgical intervention of the ACL-injured knee (ACLD) and uninjured contralateral knees (intact), after the temporary fixation of the isolated ACL grafts (ACLR), and subsequent to the temporary fixation of both the ACL and ALL grafts (ACLR + ALLR) were measured. After the measurement of ACLR, re-fixations of the ACL grafts were performed to avoid loosening grafts in the measurement of ACLR + ALLR. The Rolimeter® arthrometer (Aircast; DJO Global, USA) was used to measure the anterior tibial translation (ATT; mm) by performing the Lachman test with the manual tension at maximum. To determine instability in the pivot shift, an inertial sensor (MVP-RF8-BC; MicroStone Corporation, Japan), which contains a 3-axis accelerometer and 3-axis gyroscope, was used to measure acceleration (m/s^2^) and external rotational angular velocity (ERAV; degree/s) when performing the pivot-shift test. The high intra-observer ICC of quantitative measurements of instability in the pivot-shift test using an inertial sensor have been reported [20]. The procedures of quantitative measurements were performed according to a previous report [13]. With respect to 3-axis accelerations, the high- and low-pass filters were set at 0.5 Hz and 100 Hz, respectively, to eliminate gravity (MVPVD-S; MicroStone Corporation, Japan). Overall acceleration was calculated using the following formula |a|= √(ax2 + ay2 + az2) from 3-axis accelerations. Data from the first and last three of nine pivot-shift tests were excluded, and the average of the peak values of overall acceleration as well as the ERAV in the middle three pivot-shift tests was indicated as the evaluation values of acceleration and the ERAV. Petrigliano et al. [23] reported that external tibial rotation integrated from the ERAV during pivot shift motion was strongly correlated with potentiometer measurements of external tibial rotation. Therefore, the ERAV was considered as a representative value of tibial rotation during the reduction of tibial subluxation in the pivot-shift test.

### Assessment of the effect of additional ALL reconstruction

The ratio of ACLR to ACLR + ALLR (ACLR/ACLR + ALLR) was calculated to evaluate the effect of the addition of ALL reconstruction on acceleration and the ERAV. An ACLR/ACLR + ALLR of > 1 indicated the effect of reducing instability by additional ALL reconstruction after ACL reconstruction. It was considered that the higher value of ACLR/ACLR + ALLR, the higher the effect of reducing instability by additional ALL reconstruction. Further, subjective pivot-shift grading was performed after temporary fixations for subjective evaluation of rotational instability.

### Statistical analysis

Quantitative variables were expressed as medians and interquartile ranges (IQR). Statistical significance was set at a *P*-value of < 0.05. The nonparametric Friedman test was used to compare ACLD, intact, ACLR, and ACLR + ALLR. For post hoc analyses, Bonferroni adjustment was used. To evaluate factors in which an additional ALL reconstruction was effective for reducing instabilities, correlations were calculated using Spearman’s rank correlation coefficient to compare ACLR/ACLR + ALLR and demographic data (sex, age, time from injury to surgery, medial and lateral posterior tibial slope measured by the previously reported method using magnetic resonance imaging (MRI) [11] at the time of surgery), surgical data (medial meniscal injury without repair, and lateral meniscal injury), and quantitative measurement data adjusted for individual differences in original instability (the side-to-side difference of ATT before operation, side-to-side ratio of acceleration, and ERAV before operation (ACLD/intact), and after fixation of ACL grafts (ACLR/intact)). Statistical analyses were performed using EZR (Saitama Medical Center, Jichi Medical University, Japan), which is a graphical user interface for R (version 2.13.0; The R Foundation for Statistical Computing, Austria). More precisely, it is a modified version of R commander (version 1.6–3) designed to add statistical functions frequently used in biostatistics. A post-hoc power analysis was performed using G*Power Version 3.1.9.4. An effect size of 0.56 was calculated based on the acceleration between ACLR and ACLR + ALLR. With an underlying effect size and alpha error of 0.01, a power of 0.95 was calculated.

## Results

The included patients were 11 male and 7 female patients with a median (IQR) age of 24 (19–35) years and median time from injury to surgery of 9 (4–31) months. Two patients had knees with hyperextension of ≥ 5° preoperatively. Seventeen medial meniscal injuries were detected and comprised eight ramp lesions, two longitudinal tears in posterior segments, two bucket handle tears, and five degenerative tears in which two partial meniscectomy or three no treatment was performed. Six lateral meniscal injuries were detected and comprised 5 longitudinal tears in posterior segments and one posterior root tear. The median medial and lateral posterior tibial slopes were 6.1 (3.7–8.5) degrees and 6.4 (4.6–9.5) degrees, respectively. A negative pivot shift in subjective evaluation, which was indicated as grade 0, was confirmed in all cases after temporary fixations of the isolated ACL grafts and both the ACL and ALL grafts.

### Instability in the lachman test

**Fig. 2:**
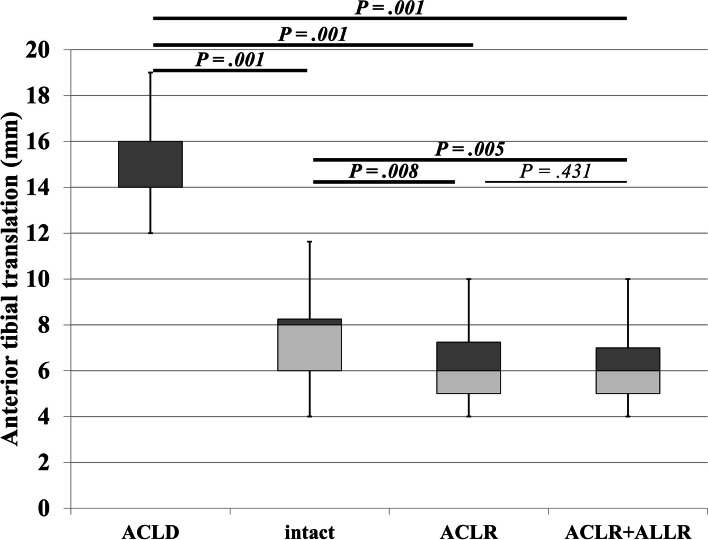
Instability in the Lachman test. Box plot of anterior tibial translation in the Lachman test. *P*-values in bold indicate statistically significant correlations (*P* < .05). ACLD, before operation for ACL-injured knee; intact, before operation for uninjured contralateral knee; ACLR, after temporary fixation of the isolated ACL grafts; ACLR + ALLR, after temporary fixation of both ACL and ALL grafts

The ATT of ACLD, intact, ACLR, and ACLR + ALLR are shown in Fig. 2. For all patients, ACLR and ACLR + ALLR were equal or lower than the intact with a statistically significant difference indicated as over-constrain due to either reconstruction of the isolated ACL or the addition of ALL. Moreover, there were no statistically significant differences between ACLR and ACLR + ALLR.

### Instability in the pivot-shift test

**Fig. 3:**
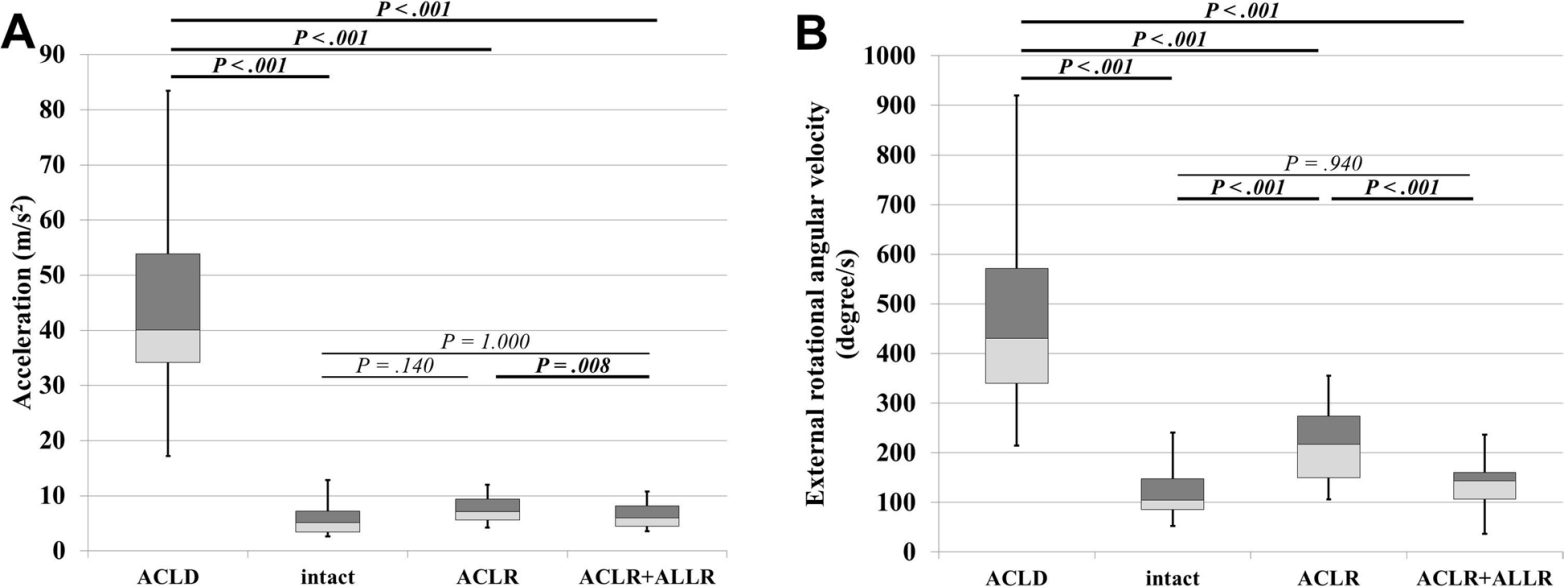
Instability in the pivot-shift test. Box plot of **(A)** acceleration and **(B)** external rotational angular velocity in the pivot-shift test. Bolded P values indicate statistically significant correlations (*P* < .05). ACLD, before operation for ACL-injured knee; intact, before operation for uninjured contralateral knee; ACLR, after temporary fixation of the isolated ACL grafts; ACLR + ALLR, after temporary fixation of both ACL and ALL grafts

The acceleration of ACLD, intact, ACLR, and ACLR + ALLR are shown in Fig. 3A. There were no statistically significant differences between intact and ACLR, or ACLR + ALLR, as indicated by the restoring stabilities of either reconstruction of isolated ACL or additional ALL. Nonetheless, for all patients, ACLR + ALLR was significantly lower than ACLR.

The ERAV of the ACLD, intact, ACLR, and ACLR + ALLR are shown in Fig. 3B. For all patients, ACLR was significantly higher than the intact, as indicated by the residual instability after isolated ACL reconstruction. Moreover, for all patients, ACLR + ALLR was significantly lower than ACLR, while there were no statistically significant differences between intact and ACLR + ALLR.

### The effect of additional ALL reconstruction

All patients had an ACLR/ACLR + ALLR ratio > 1 for both acceleration and the ERAV. Median (IQR) ACLR/ACLR + ALLR for acceleration and ERAV were 1.2 (1.0–1.5) and 1.4 (1.3–2.0), respectively. Correlations with ACLR/ACLR + ALLR are shown in Table 1. There were moderate correlations between ratios indicating greater ACLR/ACLR + ALLR for acceleration and greater ACLD/intact (R = 0.606, *P* = 0.009), and greater ACLR/intact (R = 0.503, *P* = 0.035) for acceleration, as significant factors in which an additional ALL reconstruction was effective for reducing instabilities.

**Table 1:** Correlation with ACLR/ACLR + ALLR for acceleration or the ERAV ^a^

	ACLR/ACLR + ALLR of acceleration		ACLR/ACLR + ALLR of ERAV	
	R	*P*-value	R	*P*-value
Sex	-0.055	.829	-0.297	.232
Age, years	-0.396	.103	-0.337	.171
Time from injury to surgery, month	-0.086	.735	-0.359	.143
Medial meniscal injury without repair	0.203	.419	0.108	.671
Lateral meniscal injury	-0.210	.402	-0.121	.633
Posterior tibial slope, degree				
Medial	0.146	.564	-0.033	.896
Lateral	0.248	.321	-0.179	.478
Side-to-side difference of ATT before operation, mm	0.302	.223	-0.118	.640
Acceleration				
ACLD/intact	0.606*	.009*	-0.104	.680
ACLR/intact	0.503*	.035*	0.135	.592
ERAV				
ACLD/intact	0.344	.163	0.430	.076
ACLR/intact	0.420	.084	0.267	.282

*ACL* anterior cruciate ligament, *ALL*, anterolateral ligament, *ACLR/ACLR + ALLR* ratio from the value after temporary fixation of the ACL graft to the value after temporary fixation of both ACL and ALL grafts, *ERAV* external rotational angular velocity, *R* correlation coefficient, *ATT* anterior tibial translation, *ACLD/intact* side-to-side ratio of injured to uninjured knees before operation, *ACLR/intact* side-to-side ratio of injured sides after fixation of ACL grafts to uninjured sides

^*^Statistically significant correlations (*P* < .05)

^a^ Spearman’s rank correlation coefficient was used

## Discussion

The most important finding of the present study was that ALL reconstruction added to the primary double-bundle ACL reconstruction for patients with preoperative grade 3 subjective pivot shift improved residual instability in the pivot shift, and resulted in restoring stability of the knees at the time of surgery. These findings were supported by following evidence: (1) the ERAV of ACLR were higher than intact, while (2) both acceleration and the ERAV of ACLR + ALLR were lower than ACLR and there were no significant differences between ACLR + ALLR and intact. These findings validate the hypothesis of this study.

Patients with greater instability in the pivot-shift test have been reported to have higher risk for residual instability after ACL reconstruction at the time of surgery [13]. It has been proposed that intraoperative instability in the pivot-shift test after ACL reconstruction may worsen rather than improve during follow-up, because the anteroposterior translation has been reported to increased further [3]. Therefore, even a slight residual instability, detectable only by quantitative measurement, may be undesirable thus it is valuable that ALL reconstruction can improve this residual instability. The present study has suggested that isolated double-bundle ACL reconstruction for patients with preoperative grade 3 subjective pivot shift may be not sufficient regarding of restoring stability. The effect of additional ALL reconstruction during follow-up should be investigated by quantitative evaluation in a future study.

The effect of ALL reconstruction has been reported in biomechanical cadaveric studies. It has been reported that the ERAV in the pivot-shift test after concomitant ACL and ALL reconstruction was lower than isolated ACL reconstruction for both the ACL and anterolateral structure injured knee [31]. Nitri et al. [22] reported that the internal rotation of the tibia during a simulated pivot-shift test after a combined ACL and ALL reconstruction was lower than following an ACL reconstruction alone, while the ATT was not affected by additional ALL reconstruction. Moreover, isolated ACL reconstruction was not able to restore the stability of the knee, resulting in a significant increase in residual internal rotation laxity. These results indicating that not ATT, but the rotational instability was controlled by additional ALL reconstruction is supported by the findings in the present study. Furthermore, Ferretti et al. [6] has demonstrated abnormalities of the ALL in the majority of acutely ACL-injured knees, and Saithna et al. [24] has reported that ALL has limited intrinsic healing potential, with only 30.3% healing by 12 months after ACL reconstruction. It is not possible for studies using cadaveric knees to reproduce the actual ACL injured knees with soft tissue and capsular injuries that are difficult to detect and evaluate in clinical settings. The present study therefore has an advantage in this regard.

Ueki et al. [29] reported that additional anterolateral structure augmentation, which is a similar procedure to the ALL reconstruction performed in the present study, further improved acceleration in the pivot-shift test during surgery when compared to ACL reconstruction alone using a single-bundle bone-patellar tendon-bone autograft. Although this report supports the findings of the present study, patients in the study sample included at least one revision case, cases of preoperative high-grade subjective pivot shift (9/15 cases), or knee hyperextension, which differed from the patients included in the present study. Although several additional procedures to augment anterolateral structure [4, 25] have been reported, optimal indications of these procedures are unclear. The results of the present study can contribute to the evaluation of the optimal indications for the addition of ALL reconstruction to ACL reconstruction.

The ratios relative to the intact for acceleration, indicated as ACLD/intact and ACLR/intact, were identified as factors for which additional ALL reconstruction was effective to reduce knee instability based on the results of the correlation analysis with ACLR/ACLR + ALLR. A previous study [13] showed a positive correlation between ACLD/intact and ACLR/intact for acceleration, and a cut-off value for ACLD/intact as a risk factor for the residual acceleration after fixation of ACL grafts was detected. These results suggest that quantitative evaluation of ACLD/intact for acceleration is important to evaluate both the possibility of residual acceleration after isolated ACL reconstruction and the effectiveness of additional ALL reconstruction.

The present study had some limitations: (1) Quantitative measurements were performed in a non-blinded manner with knowledge of whether the knee was uninjured or ACL-injured, or whether the knee was preoperative, post-fixation of ACL graft, or both the ACL and ALL grafts condition. (2) MRI for the injuries of the anterolateral structure in each knee was not evaluated. A three-dimensional MRI technique has been reported to be useful for the evaluation and identification of the ALL [19], while it is controversial that the ALL can be reliably identified in standard MRI techniques [5, 6, 26]. Because all included patients in the present study underwent only standard MRI, the evaluation data of MRI for the ALL tears were not included. Future studies using three-dimensional MRI and quantitative measurements may be required. (3) It is unclear whether the addition of ALL reconstruction affected postoperative clinical outcomes in this study, i.e. 2 year patient reported outcomes and objective laxity outcome. However, the correlation between instability in the pivot-shift test and functional outcomes [2] suggests it is highly likely that residual instability leads to poorer outcomes. Future studies investigating postoperative clinical results and changes in instability over time are required. (4) This study was a small cohort (eighteen patients). However, a post-hoc power analysis showed an effective power calculated. (5) Finally, instability in the pivot-shift test was measured by manually performing of the pivot-shift test, which has been reported to be less reliable than the mechanised pivot-shift test [21].

## Conclusions

The addition of ALL reconstruction to primary double-bundle ACL reconstruction for patients with preoperative grade 3 subjective pivot shift improved the residual instability in the pivot shift, resulting in the restoration of knee stability during surgery. Isolated double-bundle ACL reconstruction for patients with preoperative grade 3 subjective pivot shift should be chosen with care because it may cause residual instability. Moreover, greater instability of the pivot shift before the surgical procedure and after isolated ACL reconstruction during surgery may predict the effectiveness of an additional ALL reconstruction for improving instabilities of the pivot shift.

## Abbreviations

ACL: Anterior cruciate ligament
ALL: Anterolateral ligament
ATT: Anterior tibial translation
ERAV: External rotational angular velocity
IQR: Interquartile ranges
MRI: Magnetic resonance imaging
